# Progressive brain atrophy in Parkinson's disease patients who convert to mild cognitive impairment

**DOI:** 10.1111/cns.13188

**Published:** 2019-07-06

**Authors:** Cheng Zhou, Xiao‐Jun Guan, Tao Guo, Qiao‐Ling Zeng, Ting Gao, Pei‐Yu Huang, Min Xuan, Quan‐Quan Gu, Xiao‐Jun Xu, Min‐Ming Zhang

**Affiliations:** ^1^ Department of Radiology The Second Affiliated Hospital, Zhejiang University School of Medicine Hangzhou China; ^2^ Department of Neurology The Second Affiliated Hospital, Zhejiang University School of Medicine Hangzhou China

**Keywords:** cerebrospinal fluid, dopamine transporter, mild cognitive impairment, Parkinson's disease magnetic resonance imaging

## Abstract

**Aims:**

Cognitive impairment is a common symptom in the trajectory of Parkinson's disease (PD). However, the pathological underpinning is not fully known. We aimed to explore the critical structural alterations in the process of cognitive decline and its relationships with the dopaminergic deficit and the level of related cerebrospinal fluid (CSF) proteins.

**Methods:**

Ninety‐four patients with PD and 32 controls were included in this study. Neuropsychological tests were performed at baseline and after 28 months to identify which patients had normal cognition and which ones developed PD‐MCI after follow‐up (“converters”). Gray matter atrophy was assessed in cross‐sectional and longitudinal analyses, respectively. The associations between altered GMV with dopamine transporter (DAT) results and the level of CSF proteins were assessed.

**Results:**

Among the 94 patients with normal cognition at baseline, 24 (mean age, 63.1 years) developed PD‐MCI after 28 months of follow‐up, and 70 (mean age, 62.3 years) remained nonconverters. The converters showed significant right temporal atrophy at baseline and extensive atrophy in temporal lobe at follow‐up. Progressive bilateral frontal lobe atrophy was found in the converters. Baseline right temporal atrophy was correlated with the striatal dopaminergic degeneration in the converters. No correlation was found between the right temporal atrophy and the alterations of CSF proteins.

**Conclusion:**

Early atrophy in temporal lobes and progressive atrophy in frontal lobes might be a biomarker for developing multidomain impairment of cognition and converting to PD‐MCI. Furthermore, cognition‐related temporal atrophy might be associated with dopaminergic deficit reflected by DAT scan but independent of CSF proteins in patients with PD who convert to PD‐MCI.

## 1 INTRODUCTION

Parkinson's disease (PD) is a complex multisystem neurodegenerative disease with 6.2 million people affected.^1^, ^2^ Nonmotor symptoms are increasingly recognized in patients with PD, and cognitive decline is one of the common nonmotor symptom, which greatly worsens life quality.^3^ Therefore, investigating the mechanism of cognitive impairment could contribute to a better understanding of PD.

Studies showed that a substantial percentage of PD patients developed dementia over time.^3^, ^4^, ^5^ It is known that the presence of mild cognitive impairment (PD‐MCI) was associated with a high risk of progressing to dementia. Exploring the brain structural differences between PD‐MCI and PD‐NC is of great importance. Previous studies revealed that PD‐MCI patients had more atrophy than PD‐NC in the frontal and temporal cortex and hippocampus measured by high‐resolution magnetic resonance imaging (MRI).^6^, ^7^, ^8^ However, the consistency between these studies is poor. In a recent cross‐sectional study, temporal cortical thinning was observed in the patients who converted to PD‐MCI (the converters) before their conversion.^9^ By focusing on longitudinal observation of subcortical volume, progressive atrophy in the thalamus, caudate, and accumbens was found in the converters.^10^ Therefore, along with the cognition evolution, structural atrophy could be a potential metric for explaining cognitive impairment in PD, though currently very few studies are available to validate such findings. To better understand the mechanism of cognitive impairment in PD, researches exploring longitudinal brain structural alterations throughout the cognition evolution are needed.

Robust studies have shown that changes in neurotransmitters and pathological substances contribute to the cognitive decline in patients with PD. Dopamine transporter (DAT) imaging studies have shown that the decline in dopaminergic activity in mesolimbic and mesocortical regions is associated with the decline in cognitive performance.^3^ Alpha‐synuclein (α‐syn), amyloid plaque (Aβ), and tau pathology were significant contributors to cognitive decline in patients with PD, and the combination of them might have an additive effect.^3^, ^11^, ^12^ Specifically, the loss of neurons in the cognition‐related cortex, for example, temporal and parietal cortex, was associated with the density of Lewy body which is mainly composed of pathological α‐syn.^13^, ^14^ Only two studies explored the associations between cerebrospinal fluid (CSF) proteins and brain structures. Total α‐syn in CSF was significantly correlated with cortical‐thickness in right superior frontal in PD patients without dementia.^15^ CSF Aβ and tau were associated with gray matter volume (GMV) in temporal lobe in PD patients with dementia and all PD patients, but no significant correlations between CSF proteins and GMV were found in PD patients without dementia.^16^ Though the aggregation of pathological proteins and the depletion of dopamine could have influence on brain structure in PD, the exact relationships among them are not known, in particular, their relations in the converters and the nonconverters.

In the present study, we aimed to clarify the following questions: (a) progressive structural alterations in patients who convert to PD‐MCI, (b) the roles of dopaminergic system and CSF proteins in structural atrophy associated with cognitive impairment. Cross‐sectional and longitudinal analyses were performed to detect the gray matter (GM) atrophy in the converters. Correlation analyses were performed between altered GMV and DAT and CSF protein results.

## 2 METHODS

### 2.1 Participants

**Figure 1:**
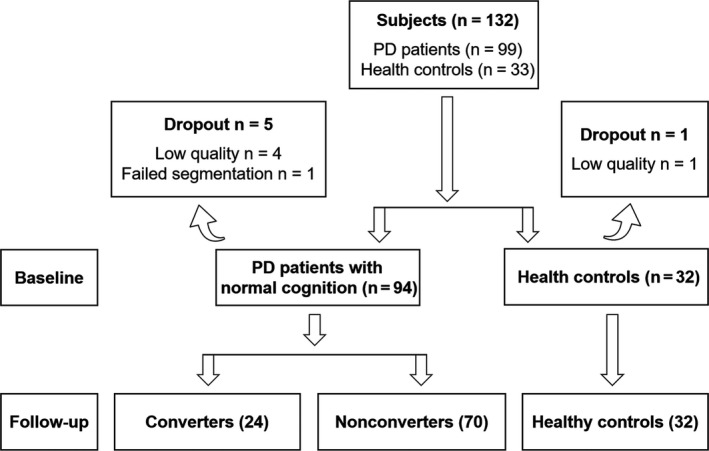
Flowchart converters: patients with PD who convert to PD‐MCI at follow‐up. Nonconverters: PD patients with stable cognition over mean 28 months

All participants included in the present study were enrolled in the Parkinson progression marker initiative (PPMI, 2011, http://www.ppmi-info.org/data; accessed in October 2018). For up‐to‐date information on the study, visit http://www.ppmi-info.org. Patients were required to meet the diagnostic criteria of PD.^17^ Healthy controls were required to have the Montreal cognitive assessment (MoCA) scores > 26 and without detectable dopaminergic deficit on DAT scan. Ninety‐nine patients with PD and 33 healthy controls with complete cognitive assessment were included in this study. Only participants passed quality control after image preprocessing were included in the final analysis (the methods for quality control were mentioned in the section of MRI preprocessing). Five patients with PD and one healthy control with poor image quality were excluded. Finally, 94 patients with PD and 32 healthy controls were included in this study (Figure 1).

### 2.2 Clinical and neuropsychological assessment

Motor symptom assessments were performed using part III of the Movement Disorder Society Unified Parkinson's Disease Rating Scale (MDS‐UPDRS III)^18^ and Hoehn and Yahr (H‐Y) staging in the “drug‐off” state. In addition, since dopaminergic drugs could have some influence on cognitive performance, we assessed the levodopa equivalent daily dose (LEDD) for each patient. Detailed methods for calculating LEDD in PPMI database were described in the PPMI manual (http://www.ppmi-info.org/).

According to the PPMI protocol, the DAT scan of the participants was collected in single‐photon emission computed tomography/computed tomography (SPECT/CT) scanner. [123I]FP‐CIT was used as imaging agent. Then, DAT images were sent to Institute for Neurodegenerative Disorders for processing and calculation of striatal binding ratios (SBRs). A Hermes Medical Solutions system for iterative reconstruction was used to process SPECT raw projection data. Iterative reconstruction was performed, and the reconstructed files were transferred to the PMOD (PMOD Technologies) for subsequent processing. After attenuation correction, Gaussian 3D 6.0‐mm filter, these files were normalized to the standard Montreal Neurologic Institute (MNI) space. The highest striatal uptake in axial slice was identified, and the 8 hottest striatal slices around it were averaged to create a single slice image. Count densities for left and right caudate and putamen as well as occipital cortex (reference tissue) were extracted. The SBRs for each of the four striatal regions were calculated as follows: SBRs = (striatal region)/(occipital cortex). Detailed procedures on how SPECT acquisition, processing, and analysis can be found online (https://www.ppmi-info.org/access-data-specimens/download-data/) and published documents.^19^, ^20^

Cerebrospinal fluid specimens were gathered by lumbar puncture within a month of MRI scan from the participants. Aβ_42_, α‐syn, and phosphorylated tau protein (p‐tau) in CSF were assessed in this study. Briefly,^21^ 15 to 20 mL of CSF was centrifuged at room temperature and followed by immediate freezing on dry ice. The CSF specimens were sent to the PPMI Biorepository Core laboratories and stored at −80°C. Aβ42 and P‐tau were analyzed using the multiplex Luminex xMAP platform in duplicate in each run, and the result was defined as the average of the concentration of duplicates. CSF α‐syn was analyzed using a commercially available enzyme‐linked immunosorbent assay kit. Detailed information about CSF proteins was described in the PPMI biologics manual (http://www.ppmi-info.org/).

Neuropsychological tests used in this study were included the following: (a) Visuospatial function was assessed using the 15‐item version of the Benton Judgment of Line Orientation test (BJLO); (b) memory was evaluated using revised scale of Hopkins Verbal Learning Test (HVLT‐R: total immediate recall and delayed recall); (c) attention and working memory were evaluated using Symbol Digit Modalities Test (SDMT) and Letter‐Number Sequencing (LNS); and (d) executive function was assessed using the semantic fluency test (SF). The neuropsychological tests were conducted during off state. We followed the PPMI diagnosis criteria in accordance with the previous documents.^22^, ^23^ The diagnosis criteria of PD‐MCI in the PPMI project resembled the Level I MDS‐Task Force classification.^24^ In detail, PD‐MCI is defined as (a) any two or more of the following cognitive tests are >1.5 SD below the standardized mean: HVLT‐R total recall ≤ 35, HVLT‐R delayed recall ≤ 35, BJLO ≤ 6, LNS ≤ 6, SF ≤ 35, SDMT ≤ 35; (b) no functional impairment which damaged daily activities seriously. Those who had normal cognition at baseline but converted to PD‐MCI after follow‐up were classified as the converters (n = 24). Patients with normal cognition at baseline and kept cognitively intact during follow‐up were defined as the nonconverters (n = 70).

### 2.3 MRI acquisition

All 3D T1‐weighted imaging were acquired on Siemens 3.0 T scanners. The scanning parameters were as follows: repetition time (TR) = 2300 ms; echo time (TE) = 2.98 ms; inversion time = 900 ms; flip angle = 90°; slice number = 176; acquisition matrix = 240 × 256 and voxel size = 1 × 1 × 1 mm^3^.

### 2.4 MRI preprocessing and analysis

VBM was performed by using the CAT12 Toolbox (Computational Anatomy Toolbox; http://dbm.neuro.uni-jena.de/cat12/) running within SPM12 (Statistical Parametric Mapping) software (http://www.fil.ion.ucl.ac.uk/spm).

The CAT12 Toolbox provided a batch for longitudinal data processing (http://www.neuro.uni-jena.de/cat12/CAT12-Manual.pdf). (a) An inverse‐consistent realignment and bias correction between the two time points were performed to calculate the mean image of the two realigned images. (b) Based on the segmentations of the mean image, spatial normalization parameters were estimated with the help of a Diffeomorphic Anatomical Registration Through Exponential Lie algebra normalization. (c) Next, these normalization parameters were applied to segmentation and modulation of the images of both times points. Then, all images of both time points were segmented into GM, white matter (WM), and CSF components and spatial normalized images were modulated to ensure the relative GMV. (d) Then, display one slice for all images was performed to check the quality of spatial registration. (e) Total intracranial volume (TIV) was estimated, and mean correlation, Mahalanobis distance, and weighted overall image quality algorithms were used to quantify image quality. If the image quality was lower than twice the standard deviation, the origin data will be checked. The images had artifacts, abnormal ventricular enlargement, and the brain tissues absent were excluded (five patients with PD and one healthy control). (f) Finally, segmented GM images were smoothed by using 8 mm full‐width‐half‐maximum isotropic Gaussian kernel.

### 2.5 Statistical analysis

Statistical analysis was performed using IBM SPSS Statistics 23.0 software. One‐sample Kolmogorov‐Smirnov test was used to test the distribution of continuous variables for normality. Normally distributed continuous variables were assessed using independent sample *t* test or one‐way analysis of variance (ANOVA). Post hoc tests were performed after ANOVA. Nonparametric data were assessed using Wilcoxon rank‐sum test (duration) and Kruskal‐Wallis test. We considered a two‐tailed *P*‐value of <0.05 as significant for each test statistic.

One‐way analysis of covariance (ANCOVA) was performed to assess the difference in GMV among three groups at baseline and follow‐up separately. Gaussian Random Field (GRF) correction (voxel *P* = 0.001, cluster *P* = 0.05) was used for multivoxel comparisons. Since Data Processing & Analysis for Brain Imaging (DPABI) is a useful toolbox that provides multiple comparisons over group pairs,^25^ the post hoc t tests were conducted using DPABI to assess the differences between each pair of groups (voxel *P* = 0.001, cluster *P* = 0.05, GRF corrected). And Bonferroni correction was used for multiple comparison correction in multiple tests. Age, sex, TIV, and UPDRS III scores were incorporated as covariates.

Longitudinally, the interaction effects of GMV alteration were determined using full‐factorial model with three groups and two time points (3×2 ANCOVA) (https://www.fil.ion.ucl.ac.uk/spm/software/spm12). And post hoc test was conducted to identify the direction of GMV alteration (converters < nonconverters, converters < healthy controls, nonconverters < healthy controls; voxel *P* = 0.001, cluster *P* = 0.05, GRF corrected). Age, gender, TIV, and UPDRS III scores were included as covariates.

Every brain region showed significant difference among three groups was saved as a mask, and then, this mask was used to extract the mean GMV of every individual using the ROI Signal Extractor tool of DPABI.^25^ Partial correlation was used to evaluate the relationship between the mean volume of region showed significant atrophy and cognitive tests, SBRs, and the level of CSF proteins in the converters and the nonconverters, respectively. Age, gender, and TIV were included as covariates. Statistical significance was set at *P* < 0.05 (false discovery rate [FDR] corrected).

## 3 RESULTS

### 3.1 Participants’ characteristics

Demographics and clinical variables at baseline were summarized in Table 1. No significant differences were observed among the converters, nonconverters, and healthy controls in age, gender, education, and scan intervals. There was no difference in disease durations and H‐Y stages between the converters and nonconverters. CSF Aβ_42_, p‐tau, and α‐syn showed no difference among three groups. Patients with PD had significantly lower SBRs in right (*P* < 0.001) and left (*P* < 0.001) caudate, right (*P* < 0.001) and left (*P* < 0.001) putamen compared to healthy controls. But the SBRs showed no difference between the converters and nonconverters. Significant difference in MDS‐UPDRS III scores was observed between two PD groups (*P* < 0.001). The converters had poorer cognitive performance in MoCA (*P* = 0.015 and 0.002, respectively) compared to the nonconverters and healthy controls at baseline. No significant difference in LEDD was found between the converters (255.25 ± 134.12) and the nonconverters (234.99 ± 163.59; *P* = 0.428). And we found no correlation between LEDD and the cognitive tests in the converters and the nonconverters.

**Table 1:** Demographics and clinical variables at baseline

	Converters	Nonconverters	Healthy controls	*P*‐value			
				A	B	C	D
Age (years)	63.12 ± 8.61	62.32 ± 8.58	59.92 ± 12.03	0.392^a^	‐	‐	‐
Gender (Male/Female)	16/8	43/27	14/18	0.156^b^	‐	‐	‐
Education (years)	14.04 ± 3.18	15.14 ± 2.91	15.22 ± 3.22	0.325^b^	‐	‐	‐
Scan interval (years)	2.61 ± 1.25	2.44 ± 1.40	2.17 ± 1.37	0.231^b^	‐	‐	‐
Duration (years)	0.92 ± 0.85	0.80 ± 0.73	‐	‐	0.907^c^	‐	‐
H‐Y stages	1.67 ± 0.48	1.68 ± 0.47	‐	‐	0.473^d^	‐	‐
MDS‐UPDRS III	23.50 ± 11.06	21.11 ± 9.34	‐	‐	0.001*,d	‐	‐
MoCA	26.04 ± 3.09	27.83 ± 1.74	28.50 ± 1.22	0.004*,b	0.015^c^, *	0.002^c^, *	0.099^c^
SBRs Caudate_R	1.74 ± 0.54	1.87 ± 0.59	2.77 ± 0.40	<0.001*,a	0.888	<0.001*,d	<0.001*,d
SBRs Caudate_L	1.75 ± 0.54	1.89 ± 0.62	2.87 ± 0.50	<0.001*,a	0.925	<0.001*,d	<0.001*,d
SBRs Putamen_R	0.73 ± 0.31	0.81 ± 0.30	2.11 ± 0.41	<0.001*,b	0.064	<0.001*,c	<0.001*,c
SBRs Putamen_L	0.76 ± 0.27	0.76 ± 0.35	2.03 ± 0.36	<0.001*,a	1.000	<0.001*,d	<0.001*,d
CSF α‐syn (pg/mL)	1687.86 ± 492.67	1709.45 ± 712.81	1892.34 ± 792.82	0.435^a^	‐	‐	‐
CSF Aβ_42_ (pg/mL)	357.53 ± 71.17	374.16 ± 102.16	376.31 ± 100.30	0.739^a^	‐	‐	‐
CSF p‐tau (pg/mL)	13.40 ± 6.04	18.19 ± 12.15	20.72 ± 14.09	0.487^b^	‐	‐	‐

Note

A = Comparison among Converters, Nonconverters and Healthy controls; B = Converters vs Nonconverters; C = Converters vs Healthy controls; D = Nonconverters vs Healthy controls.

Abbreviations: CSF, cerebrospinal fluid; L, left; MDS‐UPDRS III, part III of the Movement Disorder Society Unified Parkinson's Disease Rating Scale; MoCA, Montreal Cognitive Assessment; R, right; SBRs, striatal binding ratios.

a ANOVA.

b Kruskal‐wallis test.

c Wilcoxon rank‐sum test.

d Independent sample *T* test.

* Indicates a *P*‐value < 0.05.

Neuropsychological assessments in multiple cognitive domains were summarized in Table S1. At baseline, the converters had poorer cognitive performance in BJLO (both *P* = 0.003), SDMT (*P* = 0.005 and *P* < 0.001, respectively), and SF (*P* = 0.034 and *P* = 0.008, respectively) compared with the nonconverters and healthy controls. At follow‐up, prominently decreased cognitive scores in BJLO (*P* = 0.001 and *P* < 0.001, respectively), HVLT_immediate (both *P* < 0.001), HVLT_delayed (*P* = 0.005 and 0.001, respectively), LNS (both *P* < 0.001), SDMT (both *P* < 0.001), and SF (*P* = 0.012 and *P* = 0.002, respectively) were observed in the converters compared with that in the nonconverters and healthy controls. In addition, the converters showed significantly faster cognitive decline in BJLO (*P* = 0.002), HVLT_immediate (*P* < 0.001), and LNS (*P* = 0.002) than the nonconverters and healthy controls.

### 3.2 Cross‐sectional and longitudinal analyses of GMV

**Figure 2:**
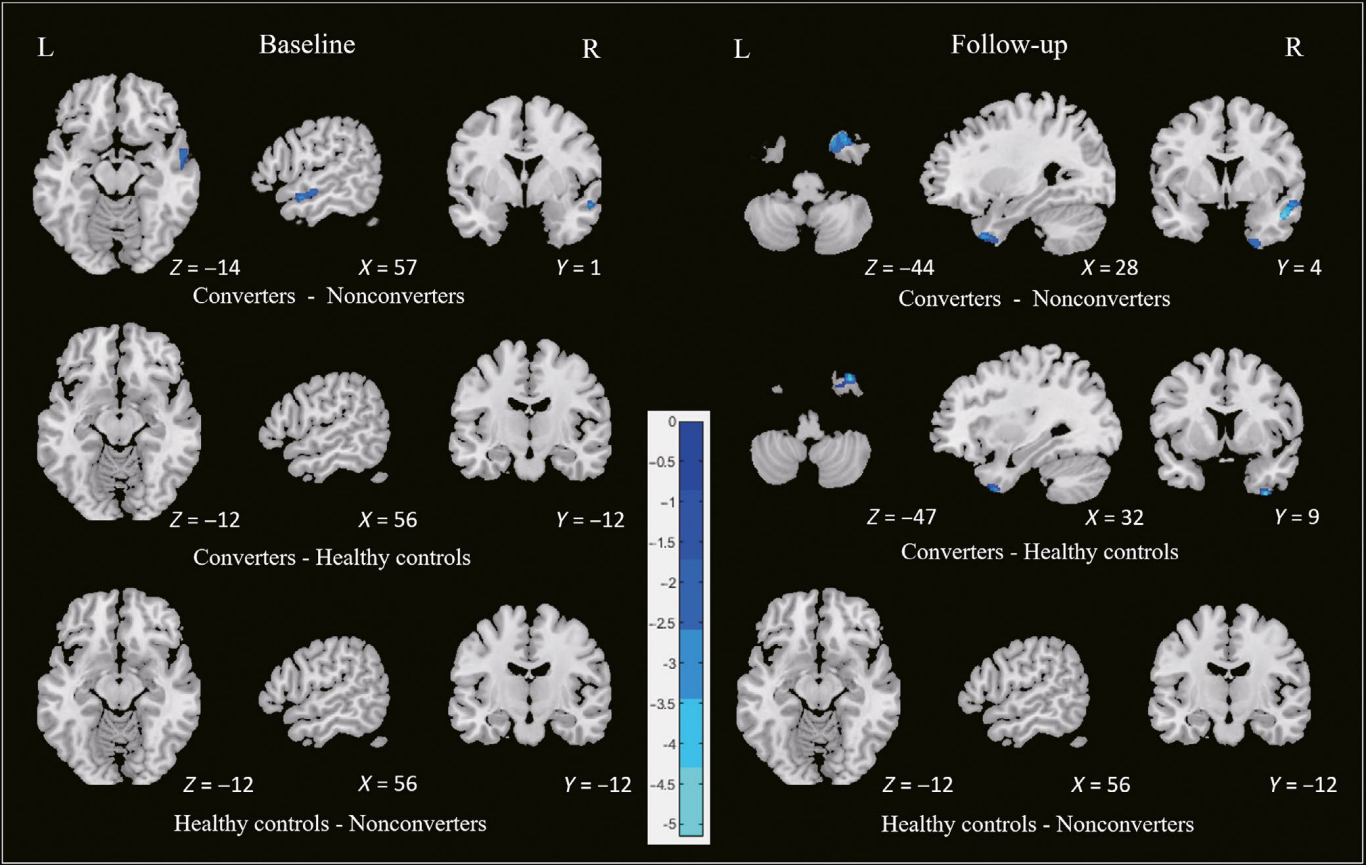
Cross‐sectional analysis among three groups at baseline and follow‐up

At baseline, there was more right temporal pole atrophy in the converters than in the nonconverters (Table 2). At follow‐up, extensive atrophy in the similar regions was found among three groups. In the converters, extensively reduced GMV relative to the nonconverters were located in right temporal poles, right middle temporal lobe. Significant right inferior temporal lobe atrophy was observed in the converters compared to healthy controls. The results of post hoc tests were reported in Figure 2 and Table 2.

**Table 2:** Anatomical locations of significant GMV alterations in the converters in cross‐sectional and longitudinal analyses

Anatomical location	Side	Peak MNI coordinate			*t*‐statistics	Cluster size
		*X*	*Y*	*Z*		
Cross‐sectional analysis (Baseline)						
The converters‐the nonconverters						
Temporal pole	R	51	6	−22	−5.09	329
Cross‐sectional analysis (Follow‐up)						
The converters‐the nonconverters						
Temporal pole	R	31	12	−46	−4.65	440
Middle temporal lobe	R	49	6	−22	−5.15	421
The converters‐healthy controls						
Inferior temporal lobe	R	34	10	−48	4.28	146
Longitudinal analysis (Interaction effect)						
The converters‐the nonconverters						
Superior frontal lobes	L, R	0	51	25	4.19	273

Abbreviations: L, left; R, right.

**Figure 3:**
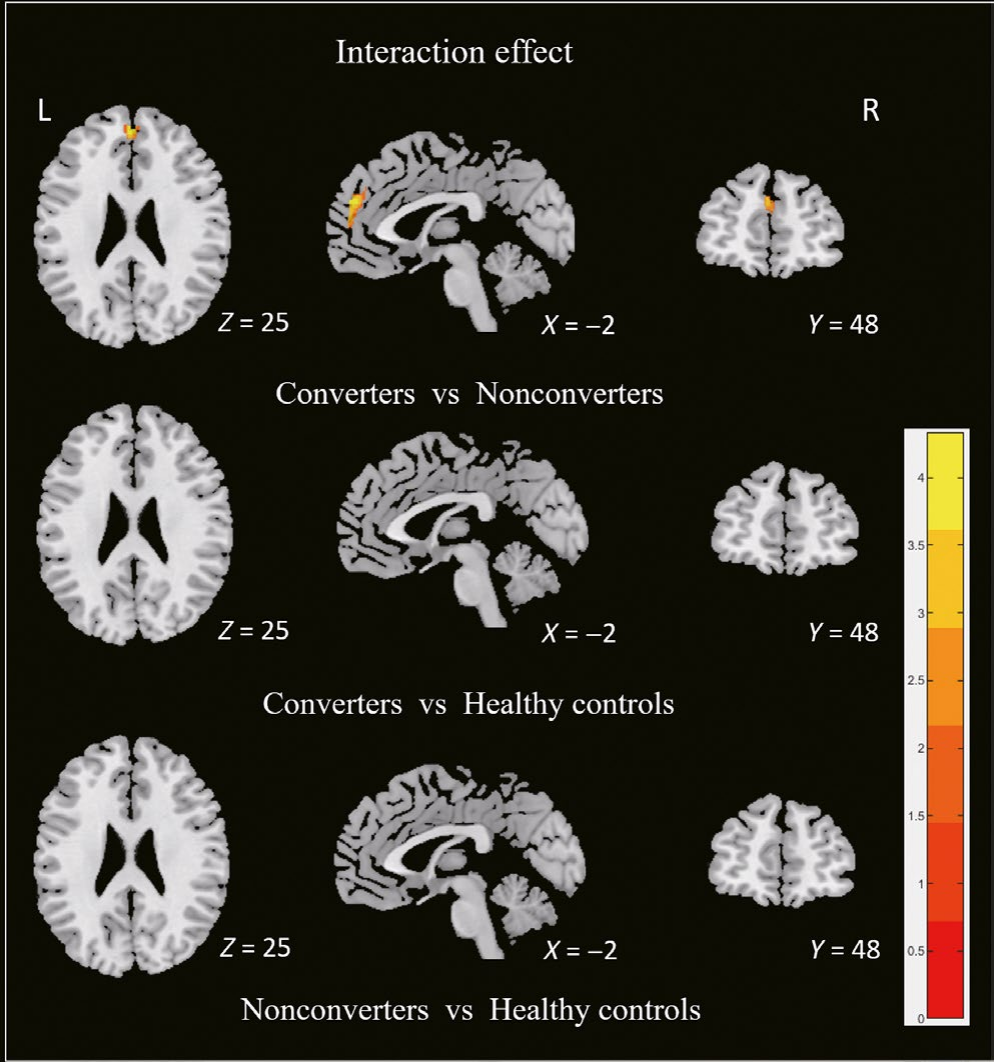
Longitudinal analysis among three groups

In longitudinal analysis, significant interaction effect was found in bilateral frontal lobes among three groups. The converters developed an increased GM atrophy in frontal lobes compared with the nonconverters during follow‐up. No significant GMV alteration was found in the nonconverters and healthy controls during follow‐up. The results of post hoc test were summarized in Figure 3 and Table 2.

### 3.3 Correlation analysis

**Figure 4:**
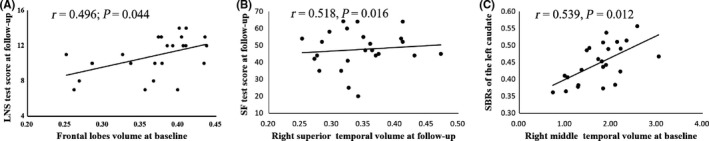
Correlation analysis. LNS: letter‐number sequencing; SF: semantic fluency; SBRs: striatal binding ratios

Correlation analyses were summarized in Figure 4. All of the correlations were found in the converters. At baseline, no correlation was found between mean volume of right temporal (region showed significant difference) and cognitive scores in the converters or nonconverters. At follow‐up, the mean volume of frontal lobes (region showed significant interaction effect between the converters and nonconverters) was positively correlated with the scores of LNS (*r* = 0.496, *P* = 0.044) in the converters (Figure 4A). The mean volume of right superior temporal (region showed significant difference between the converters and nonconverters) was positively correlated with the scores of SF (*r* = 0.518, *P* = 0.016) at follow‐up (Figure 4B). No correlation was found between the altered GMV and cognitive scores in the nonconverters.

At baseline, the mean volume of right temporal lobe (region showed significant difference between the converters and nonconverters) was correlated with the SBRs (left caudate, *r* = 0.539, *P* = 0.012) in the converters (Figure 4C) but not in the nonconverters. In addition, we did not observe significant correlation between the structural atrophy and the level of CSF proteins in the converters, the nonconverters, and even the whole PD groups.

## 4 DISCUSSION

The present study aimed to investigate the alterations of GMV in the course of cognitive degeneration by comparing the converters, nonconverters, and healthy controls. And we further explored the latent factors linking to GM atrophy. (a) The converters showed significant GM atrophy in right temporal lobe at baseline, and extensive atrophy in these regions at follow‐up compared with the nonconverters. (b) The converters showed progressive GMV atrophy in frontal lobes compared with the nonconverters during follow‐up (interaction effect). (c) In the converters group, the baseline volume in right temporal lobe showed significant atrophy was correlated with the SBRs of left caudate. (d) No correlation was found between the altered GMV and CSF proteins.

The converters showed significant right temporal atrophy at baseline and extensive atrophy in the above regions at follow‐up. And the mean volume showed significant difference was correlated with the cognitive test. Strong evidences suggested that the temporal atrophy would contribute to the dysfunction in the cognitive domains.^3^, ^26^ Moreover, Mak et al found that temporal atrophy occurred 18 months before the conversion to PD‐MCI.^9^ This highly consistent result further supports the opinion that lower temporal volume might be an important marker of the conversion to PD‐MCI in the future. However, no study further explored the progressive atrophy in the conversion of PD‐MCI. We found significant group by time interaction in superior frontal lobes which means the converters showed progressive frontal atrophy compared with the nonconverters. Though no direct study explored the progressive brain atrophy in patients who convert to PD‐MCI, two VBM studies revealed that patients with PD who converted to PD dementia had a more aggressive rate of frontotemporal atrophy.^27^, ^28^ Thus, in combination with the cross‐sectional and longitudinal investigations, early atrophy in right temporal lobe and progressive atrophy in frontal lobes could indicate the conversion to PD‐MCI.

In the converters group, the right temporal lobe atrophy was positively correlated with the decrease in SBRs in left caudate. This correlation could be explained by that the striatal dopaminergic degeneration was, to some extent, reflected by the volumetric reduction in right temporal lobe. Dysfunction of dopaminergic system plays an important role in cognitive impairment in patients with PD.^3^ It has been documented that temporal lobes are receiving dopaminergic innervation and significantly reduced dopamine receptors in these regions were found in patients with PD.^29^, ^30^ And hypometabolism in cortex (assessed using fluorodeoxyglucose—positron emission tomography) develops to histological alterations and decreased GMV in the trajectory of cognitive decline in patients with PD.^31^ Thus, our result disclosed that the disruption of dopaminergic system might reflect by the cognitive‐related temporal atrophy in patients with PD who convert to PD‐MCI in the future.

Correlation analysis was performed to clarify the effect of pathological protein on GMV atrophy. As a result, no significant correlations between the structural atrophy and CSF proteins in the converters, the nonconverters, and the whole PD groups were found. Compta et al only found positive correlations between Aβ in CSF and the GMV of temporal gyrus in PD patients with dementia and all PD patients, but no significant correlations between CSF proteins and GMV were found in PD patients without dementia.^16^ Moreover, some researches indicated that significant alterations of CSF proteins were not commonly detected among PD‐MCI, PD‐NC, and healthy controls, and significant overlaps of individual values were observed among three groups.^22^, ^32^ Thus, we speculated that changes in CSF proteins are unlikely to explain brain structural changes in early stages of PD‐MCI.

There were several limitations in the present study. First, we were using the PPMI diagnosis criteria to define PD‐MCI because the PPMI project included five cognitive tests. These diagnosis criteria resemble the Level I MDS‐Task Force classification.^7^ Though the criteria were widely used previously,^22^, ^27^ the different diagnostic criteria might bring bias in comparison. Second, the longitudinal sample size was small though it was slightly enlarged but keeping comparable to the recent works,^9^, ^10^ future works integrating neuroimaging, biochemistry, and clinical assessments are necessary to validate our findings. Third, the baseline cognitive status in the converters was not well‐matched with that in the other groups, though all three groups were diagnosed as cognitively normal, which indicated that clinical assessments should be helpful to identify patients with PD in high risk of cognitive impairments. Presently, the lower GMV found in the converters before conversion further provided objective evidences to support that the converters might have basic structural alterations. In the future, studies recruiting earlier PD patients would complement our findings. Fourth, although measurement of DAT could reflect the striatal dopamine levels in patients with PD, it cannot directly reflect the condition of dopamine release or dopamine receptor activation. In the future, technology of precision measurement should be used to directly evaluate the dopaminergic innervation and further verify our finding. Finally, it is well known that PD‐MCI is a heterogeneous condition, so that GM atrophy patterns should be further explored in different cognitive impairment subtypes.

## 5 CONCLUSION

In conclusion, early atrophy in right temporal lobe and progressive atrophy in frontal lobes might be biomarkers for developing multidomain impairment of cognition and converting to PD‐MCI. Furthermore, cognition‐related temporal atrophy might be associated with dopaminergic deficit reflected by DAT scan but independent of CSF proteins in patients with PD who convert to PD‐MCI.

## CONFLICT OF INTEREST

The authors declare no conflict of interest.

## ETHICS STATEMENT

The data included in this study were from the PPMI database. The study was approved by the Institutional Review Board or Independent Ethics Committee of all participating sites in Europe, including Attikon University Hospital (Greece), Hospital Clinic de Barcelona and Hospital Universitario Donostia (Spain), Innsbruck University (Austria), Paracelsus‐Elena‐Klinic Kassel/University of Marburg (Germany), Imperial College London (UK), Pitié‐Salpêtrière Hospital (France), University of Salerno (Italy), and in the USA, including Emory University, Johns Hopkins University, University of Alabama at Birmingham, PD and Movement Disorders Center of Boca Raton, Boston University, Northwestern University, University of Cincinnati, Cleveland Clinic Foundation, Baylor College of Medicine, Institute for Neurodegenerative Disorders, Columbia University Medical Center, Beth Israel Medical Center, University of Pennsylvania, Oregon Health and Science University, University of Rochester, University of California at San Diego, and University of California, San Francisco. Informed consent was provided according to the Declaration of Helsinki.
