# Supplementary material for: Progressive brain atrophy in Parkinson's disease patients who convert to mild cognitive impairment
Source: CNS Neurosci Ther. 2019 Jul 6;26(1):117–25. doi: 10.1111/cns.13188 (PMC6930819; doi:10.1111/cns.13188)
Supplement: Supplementary file 1 [file CNS-26-117-s001.doc]

**Supplementary Material for Review**

**Supplementary Table 1 Cognitive performance in different domains**

|  | | **Converters** | **Nonconverters** | **Healthy controls** | **P-value** | | | |
| --- | --- | --- | --- | --- | --- | --- | --- | --- |
|  |  |  |  |  | **A** | **B** | **C** | **D** |
| **BJLO** | **Baseline** | 10.59±2.65 | 12.46±2.80 | 12.71±2.82 | 0.005* ^b^ | 0.003*^c^ | 0.003*^c^ | 0.686 |
|  | **Follow-up** | 9.04±2.96 | 13.09±2.56 | 11.79±3.13 | <0.001*^b^ | <0.001* | 0.001* | 0.038*^c^ |
|  | **Change** | -1.56±2.36 | 0.56±2.63 | -0.99±2.26 | <0.001* | 0.001* | 0.407 | 0.005* |
| **HVLT_ immediate** | **Baseline** | 47.81±16.59 | 47.86±10.88 | 52.31±9.79 | 0.051 | - | - | - |
|  | **Follow-up** | 36.88±12.83 | 47.87±10.53 | 53.53±10.23 | <0.001*^a^ | <0.001* ^d^ | <0.001* ^d^ | 0.802 |
|  | **Change** | -10.83±16.30 | 0.01±9.36 | 0.60±7.69 | <0.001* | <0.001* | 0.007* | 0.493 |
| **HVLT_delayed** | **Baseline** | 50.25±16.93 | 52.28±13.38 | 57.16±9.79 | 0.011*^b^ | 0.176 | 0.005*^c^ | 0.024*^c^ |
|  | **Follow-up** | 45.00±16.35 | 51.07±9.62 | 54.81±8.90 | 0.001*^b^ | 0.005*^c^ | 0.001*^c^ | 0.044*^c^ |
|  | **Change** | -5.09±18.44 | 0.1.28±14.02 | -2.57±11.30 | 0.543 | - | - | - |
| **LNS** | **Baseline** | 10.79±2.19 | 11.99±2.34 | 12.34±3.03 | 0.088 | - | - | - |
|  | **Follow-up** | 8.96±2.88 | 11.97±2.70 | 13.00±2.73 | <0.001*^b^ | <0.001* | <0.001* | 0.881 |
|  | **Change** | -1.83±3.12 | -0.14±2.48 | 0.50±2.86 | 0.005* | 0.005* | 0.002* | 0.385 |
| **SDMT** | **Baseline** | 40.60±8.56 | 47.16±6.80 | 53.54±11.34 | <0.001*^a^ | 0.005*^d^ | <0.001* ^d^ | 0.002* ^d^ |
|  | **Follow-up** | 36.00±7.94 | 47.07±7.71 | 52.00±11.42 | <0.001*^a^ | <0.001* ^d^ | <0.001* ^d^ | 0.036* ^d^ |
|  | **Change** | -4.60±10.67 | -0.95±7.86 | -2.15±10.53 | 0.112 | - | - | - |
| **SF** | **Baseline** | 49.29±7.57 | 53.84±8.93 | 57.00±9.20 | 0.021*^b^ | 0.034*^c^ | 0.008*^c^ | 0.227 |
|  | **Follow-up** | 47.58±11.37 | 54.61±8.93 | 59.20±11.08 | 0.004*^b^ | 0.012*^c^ | 0.002*^c^ | 0.136 |
|  | **Change** | -1.71±9.27 | 0.77±8.45 | 1.80±7.62 | 0.300 | - | - | - |

BJLO: 15-item version of the Benton Judgment of Line Orientation test; HVLT_immediate: Revised Hopkins Verbal Learning Test: immediate recall; HVLT_delayed: Revised Hopkins Verbal Learning Test: delayed recall; LNS: Letter-Number Sequencing; SDMT: Symbol Digit Modalities Test; SF: Semantic fluency;

A = Comparison among Converters, Nonconverters and Healthy controls; B = Converters vs Nonconverters; C = Converters vs Healthy controls; D = Nonconverters vs Healthy controls.

^a^ ANOVA; ^b^ Kruskal Wallis Test; ^c^ Wilcoxon rank-sum test; ^d^ Independent Sample T test

*This value indicates a significant difference between groups.
